# Up-front and Salvage Transoral Robotic Surgery for Head and Neck Cancer: A Belgian Multicenter Retrospective Case Series

**DOI:** 10.3389/fonc.2017.00015

**Published:** 2017-02-09

**Authors:** Jeroen Meulemans, Christophe Vanclooster, Tom Vauterin, Emmanuel D’heygere, Sandra Nuyts, Paul M. Clement, Robert Hermans, Pierre Delaere, Vincent Vander Poorten

**Affiliations:** ^1^Otorhinolaryngology-Head and Neck Surgery, University Hospitals Leuven, Leuven, Belgium; ^2^Department of Oncology, Section Head and Neck Oncology, KU Leuven, Leuven, Belgium; ^3^Otorhinolaryngology-Head and Neck Surgery, AZ Sint-Lucas, Ghent, Belgium; ^4^Otorhinolaryngology-Head and Neck Surgery, AZ Sint-Jan, Bruges, Belgium; ^5^Radiation Oncology, University Hospitals Leuven, Leuven, Belgium; ^6^Department of Oncology, Section Experimental Radiotherapy, KU Leuven, Leuven, Belgium; ^7^Medical Oncology, University Hospitals Leuven, Leuven, Belgium; ^8^Department of Radiology, University Hospitals Leuven, Leuven, Belgium

**Keywords:** transoral robotic surgery, squamous cell carcinoma, oropharyngeal cancer, supraglottic cancer, salvage surgery

## Abstract

**Introduction/aim:**

We analyzed the functional and oncologic outcomes of primary and salvage transoral robotic surgery (TORS) procedures, performed in three Belgian institutions with a similar philosophy.

**Patients and methods:**

A total of 86 patients who underwent TORS between 24-12-2009 and 25-09-2015 were retrospectively reviewed. Descriptive statistics, overall survival (OS), disease-specific survival (DSS), and disease-free survival (DFS; Kaplan–Meier), and the variation of these outcomes according to whether patients had primary or salvage TORS were evaluated (univariate log-rank analysis).

**Results:**

Of 86 patients, 56 (65.1%) underwent TORS as a primary treatment and 30 (34.9%) as a salvage procedure for recurrent or second primary cancer. Tumor location was mainly oropharynx (*N* = 63; 73.3%) followed by supraglottic larynx (*N* = 11; 12.8%), hypopharynx (*N* = 11; 12.8%), and glottic larynx (*N* = 1; 1.2%). In the up-front TORS group, most tumors were classified as cT1 (*N* = 23; 41.1%)/pT1 (*N* = 24; 42.9%) or cT2 (*N* = 27; 48.2%)/pT2 (*N* = 27; 48.2%) and cN0 (*N* = 18; 32.1%), cN1 (*N* = 13; 23.2%), or cN2 (*N* = 25; 44.6%). In the salvage TORS group, most tumors were cT1-rT1 (*N* = 18; 60.0%)/pT1-rpT1 (*N* = 18; 60.0%) or cT2-rT2 (*N* = 12; 40.0%)/pT2-rpT2 (*N* = 7; 23.3%) and cN0 (*N* = 25; 83.3%). Neck dissection was performed in 87.5% of primary cases and 30.0% of salvage cases. In the up-front TORS group, patients were postoperatively submitted to follow-up (*N* = 13; 23.2%) or received adjuvant radiotherapy, either as single modality (*N* = 26; 46.4%) or with concomitant cisplatin (*N* = 15; 26.8%). On the other hand, most salvage TORS patients did not receive any adjuvant therapy (*N* = 19; 63.3%). Mean and median follow-up was 23.1 and 21.2 months, respectively. Functional results were excellent (no definitive tracheostomy, long-term tube feeding in 1.8% of primary cases, and 20% of salvage cases). In the up-front TORS group, estimated 2-year OS was 88.5% (SE = 5.0%), 2-year DSS was 91.8% (SE = 4.6%) and 2-year DFS was 86.1% (SE = 5.3%). In the salvage TORS group, estimated 2-year OS was 73.5% (SE = 10.9%), 2-year DSS was 93.3% (SE = 6.4%), and 2-year DFS was 75.8% (SE = 9.7%). Comparing outcome of primarily treated patients to salvage patients, a non-statistically significant trend toward better OS (*p* = 0.262) and DFS (*p* = 0.139) was observed.

**Conclusion:**

This retrospective study confirms favorable oncologic and functional outcomes of TORS for selected head and neck malignancies, both in the primary and in the salvage setting.

## Introduction

Classic open “en bloc” surgical treatment of most head and neck malignancies is often associated with considerable mutilation and functional adverse effects regarding speech, swallowing function, and respiration, leading to decreased quality of life. Since the early nineties, various “landmark studies” have assessed non-surgical treatment for advanced-stage head and neck cancer, including advanced laryngeal, hypopharyngeal, and oropharyngeal squamous cell carcinoma (SCC). These studies evaluated “organ preservation protocols,” such as induction chemotherapy and subsequent radiotherapy (RT) (1, 2) or more recently concurrent chemoradiation (CRT) (3, 4) and reported oncologic results comparable to those obtained by the classic combination of surgery and postoperative RT. These findings induced a paradigm shift away from open ablative surgery toward organ preservation protocols with curative intent over the last decades (5). It is obvious that these non-surgical treatment protocols are associated with less mutilation when compared with classic open resections of advanced head and neck malignancies. However, major functional complications (dysarthria/dysphonia, trismus, dysphagia, xerostomia, dyspnea, and stridor) are not uncommon. This implies that these organ preservation strategies do not translate into an adequate function preservation for all treated patients (6–8). Although transoral laser microsurgery (TLM) has been, mainly in Europe, a valid minimally invasive surgical technique to address selected malignancies of the upper aerodigestive tract since its introduction in the 1980s, it is the recent introduction of transoral robotic surgery (TORS), which has revived the interest to treat a selection of head and neck malignancies surgically through a minimally invasive approach. The underlying idea is that a combination of less invasive surgical removal of tumors, that allows a less toxic non-surgical adjuvant therapy, would combine a disease control rate comparable to non-surgical organ preservation strategies with more satisfying functional results. TORS for treatment of head and neck malignancies was introduced in 2006 and has become well established in recent years, especially for the resection of early-stage oropharyngeal or supraglottic SCC, yielding excellent oncologic and functional outcomes. This makes primary TORS a possible alternative to non-surgical organ preservation regimens (9–18). Also for recurrent oropharyngeal carcinoma after primary (chemo)radiotherapy, there are reports that salvage TORS may yield better functional results when compared to radical open salvage surgery (19). In this multicenter retrospective case series, we reviewed functional and oncologic outcomes of primary or up-front and salvage TORS procedures performed in three Belgian hospitals.

## Patients and Methods

### Patients

This study was approved by and carried out in accordance with the recommendations of the Institutional Review Board (University Hospital Leuven Committee for Medical Ethics: IRB study number s58234). Informed consent for retrospective studies with anonymized data is not required according to Belgian law.

All consecutive (*n* = 86) patients with malignant head and neck neoplasms who were selected to undergo TORS between 24-12-2009 and 25-09-2015 were included in this multicenter retrospective case series. TORS was performed in three Belgian medical institutions: the University Hospitals of Leuven (Leuven, Belgium), the General Hospital AZ Sint-Lucas (Ghent, Belgium), and the General Hospital AZ Sint-Jan (Bruges, Belgium). The decision to submit a patient with a head and neck malignancy to TORS always resulted from discussion during a multidisciplinary tumor board meeting. Prior to surgery, patients were, according to the tumor board advice, properly staged and screened for distant disease. Tumor stage was determined according to the Union for International Cancer Control seventh edition staging system for malignant head and neck tumors (20).

### Treatment

Transoral robotic resection of tumors was performed using either the Da Vinci S or Da Vinci Si system (Intuitive Surgical Inc., Sunnyvale, CA, USA). Transoral exposure of the tumor was achieved using the Feyh-Kastenbauer retractor (Gyrus, Tuttlingen, Germany), Crowe-Davis retractor, or the laryngeal advanced retractor system (Fentex, Tuttlingen, Germany). The Da Vinci system was equipped with a 5 mm Maryland dissector and 5 mm monopolar spatula. Resection of the tumor was performed in accordance with general oncologic principles, always aiming for a complete tumor removal. In patients with clinically positive neck nodes and in patients with high risk (≥20%) for occult nodal involvement, neck dissections (uni- or bilateral) were performed immediately before or after the TORS procedure or as a second-stage procedure. In patients who presented with a clinically and radiographically negative neck who underwent prior irradiation of the neck, a watchful waiting policy was preferred. When deemed necessary by the senior surgeons (CVC, TV, and VVP), a nasogastric (NG) feeding tube was placed after the procedure to facilitate caloric intake in the immediate postoperative phase. A tracheotomy was only performed in those cases at risk for developing breathing problems postoperatively due to airway edema. Resection specimens were oriented and sent to the pathologist. In case of oropharyngeal SCC (OPSCC), human papilloma virus (HPV) status was assessed either by p16 immunostaining or by HPV *in situ* hybridization, depending on the institution where surgery was performed. Following surgical treatment and pathologic examination of the resection specimen, the patients were rediscussed during the multidisciplinary tumor board meeting before any adjuvant therapy [RT, CRT, additional surgery, or photodynamic therapy (PDT)] was administered. Adjuvant therapy was reserved for patients with adverse pathological features, including advanced T and/or N status, positive or close surgical margins, extracapsular nodal involvement, perineural invasion, and lymphovascular invasion. After termination of treatment, regular follow-up visits were organized every 2 months during the first 2 years, every 3 months during the third year, every 4 months during the fourth year, and every 6 months thereafter. Baseline imaging of the neck (CT or MRI) was performed 3–4 months after treatment and was repeated 1 year after treatment to exclude locoregional recurrence (21). Yearly imaging of the chest (plain chest radiograph or CT chest) was performed in order to detect lung metastases.

### Data and Statistical Analysis

Anonymized data were retrospectively collected from the three participating centers and pooled in a central structured electronic database. These data were related to patient and tumor characteristics, treatment, oncologic outcomes, and functional outcomes. Data were analyzed using SPSS version 22.0 statistical software (IBM Corp., Armonk, NY, USA). Descriptive statistics were used to represent data where applicable. Fisher’s exact test with Freeman–Halton extension was used to compare margin status between the primary and salvage group in a 2 × 3 contingency table. Kaplan–Meier methods were used to estimate overall survival (OS), disease-free survival (DFS), and disease-specific survival (DSS) and univariate analysis using log-rank testing was employed to compare these survival data between subgroups (primary surgery group versus salvage surgery group). Statistical significance was defined at the *p* < 0.05 level.

## Results

### Patient Characteristics

In this multi-institutional case series, 86 patients who underwent TORS were included. The population consisted of 65 males (75.6%) and 21 females (24.4%). Mean age at the time of TORS procedure was 63.4 years. Of the 74 patients with known smoking status, 63 (85.1%) were active or former smokers, while 11 patients (14.9%) had never smoked. Concerning alcohol use, data of 73 patients were obtained. Of these patients, 55 (75.3%) were active daily drinkers, 15 (20.5%) were occasional drinkers, and 3 (4.1%) had quit drinking. Patient demographics are summarized in Table 1.

**Table 1:** **Overview of patient demographics, histology, HPV status, and transoral robotic surgery (TORS) indication**.

Characteristic	Value (*N* = 86)	%
**Age, mean (SD), years**	63 (9.7)	
**Sex**		
Male	65	75.6
Female	21	24.4
**Smoking history**		
Yes (active or past)	63	73.2
No	11	12.8
Unknown	12	14.0
**Alcohol use**		
Active abuse (≥5 U/day)	19	22.1
Daily drinker (<5 U/day)	36	41.9
Occasional drinker	15	17.4
Past abuse	3	3.5
Unknown	13	15.1
**Histology**		
Squamous cell carcinoma	83	96.5
Mucoepidermoid carcinoma	1	1.2
Mucinous cystadenocarcinoma	1	1.2
Primary laryngeal carcinoid tumor	1	1.2
**P16 staining (19 tested)**		
Positive	11	57.9
Negative	8	42.1
**HPV ISH (29 tested)**		
Positive	11	37.9
Negative	18	62.1
**TORS indication**		
Up-front (primary) TORS	56	65.1
Salvage TORS	30	34.9

*HPV, human papillomavirus; ISH, in situ hybridization*.

### Treatment Characteristics

Of the 86 patients treated with TORS, 56 (65.1%) underwent TORS as an up-front treatment and 30 (34.9%) were treated in a salvage setting for recurrent or second primary cancer. TORS for salvage treatment of a second primary head and neck cancer after the patient had been treated previously for another head and neck cancer was performed in 20 cases (66.7% of salvage cases), while 10 patients (33.3% of salvage cases) underwent TORS for salvage treatment of a local recurrence after primary surgery (*N* = 1), RT (*N* = 3), CRT (*N* = 4), or a combination of surgery and RT or CRT (*N* = 2).

Neck dissection was performed in the majority of up-front TORS cases (*N* = 49 or 87.5%), but was not frequent in salvage cases who only underwent neck dissection in case of cN + status (*N* = 9 or 30.0%). Neck dissection was most often combined with TORS in a one-stage procedure (*N* = 51) versus as a second-stage procedure (*N* = 7).

Mean hospital stay was 6.8 days (median 5 days, range 2–33 days, SD 5.9 days) in the up-front TORS group versus 9.3 days (median 7 days, range 2–67 days, SD 11.8 days) in the salvage group.

No patients received neoadjuvant treatment prior to TORS. Concerning adjuvant strategy, two patients died in the postoperative phase before any adjuvant strategy was decided upon. In the up-front TORS group, patients were postoperatively submitted to follow-up (*N* = 13; 23.2%) or received adjuvant RT, either as single modality (*N* = 26; 46.4%) or with concomitant cisplatin (*N* = 15; 26.8%). One patient underwent a combination of salvage TLM and RT. On the other hand, most salvage TORS patients did not receive any adjuvant therapy (*N* = 19; 63.3%). Adjuvant RT and CRT were administered in 4 (13.3%) and 2 (6.7%) salvage patients, respectively. Two other salvage patients (6.7%) underwent salvage surgery (TLM in one case and total laryngectomy in one case), and two other patients (6.7%) were treated with postoperative PDT because of involved resection margins after TORS procedure for recurrent SCC of the base of tongue after primary RT or CRT (22). Detailed data on neck dissection and adjuvant strategy for both the up-front and salvage groups are depicted in Tables 2 and 3.

**Table 2:** **Overview of treatment and tumor characteristics for the up-front transoral robotic surgery group (*N* = 56)**.

Characteristic	Value (*N* = 56)	%
Subsite		
Oropharynx	46	82.1
Base of tongue	9	
Tonsil	32	
Posterior wall	1	
Vallecula	2	
Glossotonsillar sulcus	1	
Retromolar trigone	1	
Supraglottic larynx	5	8.9
Epiglottis	3	
Aryepiglottic fold	1	
Unknown	1	
Hypopharynx (pyriform sinus)	5	8.9
Clinical tumor classification		
cT0 Oropharynx	1	1.8
cT1	23	41.1
Oropharynx	17	
Supraglottic larynx	3	
Hypopharynx	3	
cT2	27	48.2
Oropharynx	24	
Supraglottic larynx	2	
Hypopharynx	1	
cT3	4	7.1
Oropharynx	3	
Hypopharynx	1	
cT4a Oropharynx	1	1.8
Clinical nodal classification		
cN0	18	32.1
cN1	13	23.2
cN2	25	44.6
Neck dissection		
Yes	49	87.5
Levels I–III	4	
Levels II–III	1	
Levels II–IV	10	
Levels I–IV	4	
Levels II–V	10	
Levels I–V	20	
No	7	12.5
Surgical margin status		
Clear	21	37.5
Close (<5 mm)	10	17.9
Positive	24	42.9
Second primary in margin	1	1.8
Pathological tumor classification		
pT1	24	42.9
Oropharynx	18	
Supraglottic larynx	3	
Hypopharynx	3	
pT2	27	48.2
Oropharynx	25	
Supraglottic larynx	1	
Hypopharynx	1	
pT3	2	3.6
Oropharynx	1	
Hypopharynx	1	
pT4	2	3.6
Oropharynx	1	
Hypopharynx	1	
pR2 (macroscopic residual tumor) (oropharynx)	1	1.8
Pathological nodal classification		
pNx	7	12.5
pN0	15	26.8
pN1	11	19.6
pN2a	7	12.5
pN2b	16	28.6
pN2c	0	
Adjuvant treatment		
No adjuvant treatment, follow-up	13	23.2
Radiotherapy	26	46.4
Chemoradiotherapy	15	26.8
Surgery (TLM) + radiotherapy	1	1.8
Undetermined (early death)	1	1.8

*TLM, transoral laser microsurgery*.

**Table 3:** **Overview of treatment and tumor characteristics for the salvage transoral robotic surgery group (*N* = 30)**.

Characteristic	Value (*N* = 30)	%
Reason for salvage surgery		
Local recurrence	10	33.3
Second primary tumor	20	66.7
Subsite		
Oropharynx	17	56.7
Base of tongue	8	
Tonsil	2	
Posterior wall	3	
Vallecula	2	
Soft palate	2	
Supraglottic larynx	6	20.0
Epiglottis	5	
Aryepiglottic fold	1	
Hypopharynx (pyriform sinus)	6	20.0
Glottic larynx	1	3.3
Clinical tumor classification		
cT1/rT1	18	60.0
Oropharynx	8	
Supraglottic larynx	4	
Hypopharynx	6	
cT2/rT2	12	40.0
Oropharynx	9	
Supraglottic larynx	2	
Glottic larynx	1	
Clinical nodal classification		
cN0	25	83.3
cN1	3	10.0
cN2	2	6.7
Neck dissection		
Yes	9	30.0
Superselective neck dissection (1 or 2 levels)	3	
Levels II–V	5 (1 bilateral)	
Levels II–IV	1 (bilateral)	
No	21	70.0
Surgical margin status		
Clear	10	33.3
Close (<5 mm)	8	26.7
Positive	10	33.3
Not assessable (coagulation artifacts)	2	6.7
Pathological tumor classification		
pT0/rpT0 oropharynx	1	3.3
pT1/rpT1	18	60.0
Oropharynx	9	
Supraglottic larynx	5	
Hypopharynx	4	
pT2/rpT2	7	23.3
Oropharynx	5	
Supraglottic larynx	1	
Hypopharynx	1	
pT4/rpT4 glottic larynx	1	3.3
pTx/rpTx (not specified)	3	10.0
Pathological nodal classification		
pNx	21	70.0
pN0	3	10.0
pN1	3	10.0
pN2a	0	
pN2b	2	6.6
pN2c	1	3.3
Adjuvant treatment		
No adjuvant treatment, follow-up	19	63.3
Radiotherapy	4	13.3
Chemoradiotherapy	2	6.7
Photodynamic therapy	2	6.7
Surgery (TLM, total laryngectomy)	2	6.7
Undetermined (early death)	1	3.3

*TLM, transoral laser microsurgery*.

In the up-front TORS group, eight complications and adverse events (intraoperative, early postoperative/in-hospital, and late postoperative) were encountered. These were intraoperative accidental perforation of the aerodigestive tract eventually leading to fistula formation (*N* = 1), prolonged pain (*N* = 1), pneumonia (*N* = 1), lingual edema (*N* = 1), acute myocardial infarction (*N* = 2 of whom one patient died 10 days after TORS), and respiratory collapse (*N* = 2 of whom one patient with known COPD and restrictive lung disease due to severe scoliosis died at postoperative day 30). In the salvage group, 13 complications and adverse events were registered. Teeth abrasion was encountered intraoperatively in two patients. Early treatment-related complications and adverse events (during hospitalization) were respiratory collapse (*N* = 2 of whom one died), temporarily nasal regurgitation of food (*N* = 1), fistula formation (*N* = 1), serious dysphagia and aspiration, necessitating total laryngectomy (*N* = 1), and postoperative hemorrhage needing surgical revision (*N* = 1). Late treatment-related complications (after hospital discharge) were encountered in five salvage patients: one patient developed necrosis of the posterior oropharyngeal wall and spondylitis/spondylodiscitis of C3–C4 4 months after TORS, one patient who underwent robotic oropharyngectomy developed osteomyelitis of the clivus and right petrous apex 2 years after surgery, one patient developed late aspiration and weight loss necessitating gastrostomy tube (G-tube) placement 5 months after TORS, and two patients developed necrosis of the resection site of whom one evolved to a late carotid blowout and subsequent death (11 months after TORS). Concerning the three treatment-related, early/in-hospital deaths, a lethal myocardial infarction occurred in one patient at postoperative day 10 and would probably also have occurred if a classical surgical procedure had been carried out. The patient had undergone a thorough preoperative anesthesiologic checkup including ECG and the patient was considered medically fit for surgery. One of two patients with a fatal respiratory collapse had a salvage oro-hypopharyngectomy that did not interfere with the laryngeal airway; it was deemed that a tracheostomy was not necessary on the condition of a nil-by-mouth regimen. Although there were no signs of aspiration in the first 24 h and the patient was on intravenous antibiotics, the patient developed a fulminant atypical bilateral upper and lower lobe pneumonia, uncontrollable despite performing a tracheostomy at 24 h postoperatively, artificial ventilation, and antibiotic therapy. The patient succumbed to this evolution. In retrospect, an up-front tracheostomy may have prevented this, but the postoperative course remains difficult to explain. The second patient who died from respiratory problems had severe COPD and restrictive lung disease related to severe scoliosis. He underwent an up-front transoral resection of a small cT1 SCC of the base of tongue and because of the limited resection, no tracheotomy was performed. The patient continued to need ventilatory assistance postoperatively, eventually leading to death at postoperative day 30. Taken these cases into consideration, a thorough preoperative evaluation of the cardiorespiratory condition and low threshold for performing a tracheotomy (especially in salvage cases) are necessary for reducing unfavorable outcomes.

Treatment characteristics are presented in detail in Table 2 (*up-front TORS group*) *and* Table 3 (*salvage TORS group*).

### Tumor Characteristics

Tumor location was mainly oropharynx (*N* = 63; 73.3%) and to a lesser extent supraglottic larynx (*N* = 11; 12.8%), hypopharynx (*N* = 11; 12.8%), and glottic larynx (*N* = 1; 1.2%). Patients were preoperatively classified as cT0 (*N* = 1; 1.2%) (no apparent disease after primary excision biopsy elsewhere), cT1 (*N* = 41; 47.7%), cT2 (*N* = 39; 45.4%), cT3 (*N* = 4; 4.7%), and cT4a (*N* = 1; 1.2%). Concerning the neck, classification was cN0 in 43 patients (50.0%), cN1 in 16 patients (18.6%), and cN2 in 27 patients (31.4%). All but one N2 patients presented with unilateral nodal involvement (N2a/N2b). Patients were definitively classified as pT0/rpT0 (*N* = 1; 1.2%), pT1/rpT1 (*N* = 42; 48.8%), pT2/rpT2 (*N* = 34; 39.5%), pT3/rpT3 (*N* = 2; 2.3%), pT4a/rpT4a (*N* = 3; 3.5%), pR2 (*N* = 1, 1.2%), and pTx/rpTx (*N* = 3, 3.5%). Concerning the neck, classification was pNx in 28 patients (32.6%), pN0 in 18 patients (20.9%), pN1 in 14 patients (16.3%), pN2a in 7 patients (8.1%), pN2b in 18 patients (20.9%), and pN2c in 1 patient (1.2%). Detailed data on location, cTNM, and pTNM classification for both the up-front and salvage groups are depicted in Tables 2 and 3.

On definitive pathologic examination, the vast majority of operated head and neck neoplasms proved to be SCC (*N* = 83, 96.5%). Minor salivary gland mucoepidermoid carcinoma and mucinous cystadenocarcinoma were diagnosed each in one case. A primary laryngeal carcinoid tumor was observed in one patient with a supraglottic neoplasia. HPV status was assessed in 39 of 46 OPSCC cases treated by up-front TORS by either p16 immunostaining or HPV ISH. Of these 39 assessed tumors, 22 (56.4%) were considered HPV related. Of the 17 oropharyngeal cancers who underwent salvage surgery, HPV status was assessed in nine cases, which all proved HPV negative.

Concerning surgical margin assessment on definitive pathologic examination, clear margins were obtained in 37.5% (*N* = 21) of primary cases and 33.3% (*N* = 10) of salvage cases and close margin status was apparent in 17.9% (*N* = 10) and 26.7% (*N* = 8), respectively. However, margins were judged positive in 42.9% (*N* = 24) of primary cases and 33.3% (*N* = 10) of salvage cases, yielding a positive margin rate in the overall population of 39.5% (*N* = 34). In two salvage cases, margins were not assessable due to excessive coagulation artifacts. Interestingly, in one primary case, a small second primary tumor involved the section margins of a radically removed primary tumor. No statistically significant difference in margin status between the primary and salvage groups was observed (*p* = 0.63, Fisher’s exact test with Freeman–Halton extension). Margin status for both the up-front TORS group and salvage TORS group are illustrated in Tables 2 and 3 respectively. Positive margin status was the only adverse factor leading to the decision to apply adjuvant treatment in 18 out of 56 up-front TORS cases (32.1%).

### Functional Outcome

Data concerning necessity for feeding-tube placement were available in 85 cases. The majority of up-front TORS patients (*N* = 38, 69.1%) did not need a feeding tube (NG nor gastrostomy tube) in the immediate postoperative period. To the contrary, only 12 salvage patients (40%) were allowed to continue oral feeding after TORS. A NG tube was placed in 17 up-front cases (30.9%) and 12 salvage cases (40%), which was removed after a mean duration of 3.2 days (median 3 days, range 2–7 days, SD 1.4 days) and 5 days (median 5 days, range 1–15 days, SD 3.8 days), respectively. Removal of the NG feeding tube was determined by the ability of the patients to achieve a normal oral daily caloric intake. Four salvage patients (13.3% of salvage group) suffered from severe dysphagia in the preoperative period and were dependent on a G-tube for their daily caloric intake before TORS. All these patients were salvage cases who underwent prior head and neck surgery (*N* = 1) or a combination of surgery and adjuvant RT (*N* = 3). They all remained G-tube dependent after TORS. In the postoperative phase, two salvage patients and one up-front patient (pT3N2bM0 with adjuvant RT) developed severe dysphagia with aspiration immediately after TORS and no subsequent favorable clinical evolution, necessitating long-term G-tube feeding. As one can expect, swallowing outcomes in terms of G-tube dependency seem favorable in the up-front TORS group (1.8% G-tube dependency) when compared to the salvage group (20% G-tube dependency including G-tubes already present before TORS).

Concerning postoperative airway management, tracheotomy was deemed necessary in one up-front TORS case (1.8% of up-front population) and in seven salvage cases (23.3% of salvage population). The average duration of tracheotomy presence was 10 and 19 days (median 11 days, range 2–75 days, SD 25.1 days), respectively. The outlier (tracheotomy presence of 75 days) was a patient for whom it was decided to preserve the tracheotomy until completion of adjuvant PDT. No unforeseen tracheotomies in the postoperative phase were necessary. Main indication for tracheotomy was location of the tumor in the supraglottic larynx and base of tongue, especially in the salvage setting (five out of eight tracheotomies).

### Oncologic Outcome and Survival

Mean and median follow-up length for the total population was 23.1 and 21.2 months, respectively (range 0.2–55.1 months, SD 15.4 months). Mean and median follow-up for the population alive at the end of follow-up was 24.5 and 21.7 months, respectively (range 0.3–55.1 months, SD 15.5 months). Death occurred in 16 patients (18.6% of total population). In the up-front TORS group, death occurred in 10 patients during follow-up: two deaths resulted from early or in-hospital treatment-related complications (cf. Treatment Characteristics). Local/locoregional recurrence and distant disease were responsible for one and four deaths, respectively, and in three patients, death was considered non-disease and non-treatment related. In the salvage TORS group, six deaths were encountered during follow-up, with one death due to an early in-hospital treatment-related complication and one death due to a late treatment-related complication (cf. Treatment Characteristics). Local recurrence was responsible for two deaths and another two deaths were considered neither non-disease nor treatment related.

**Figure 1:**
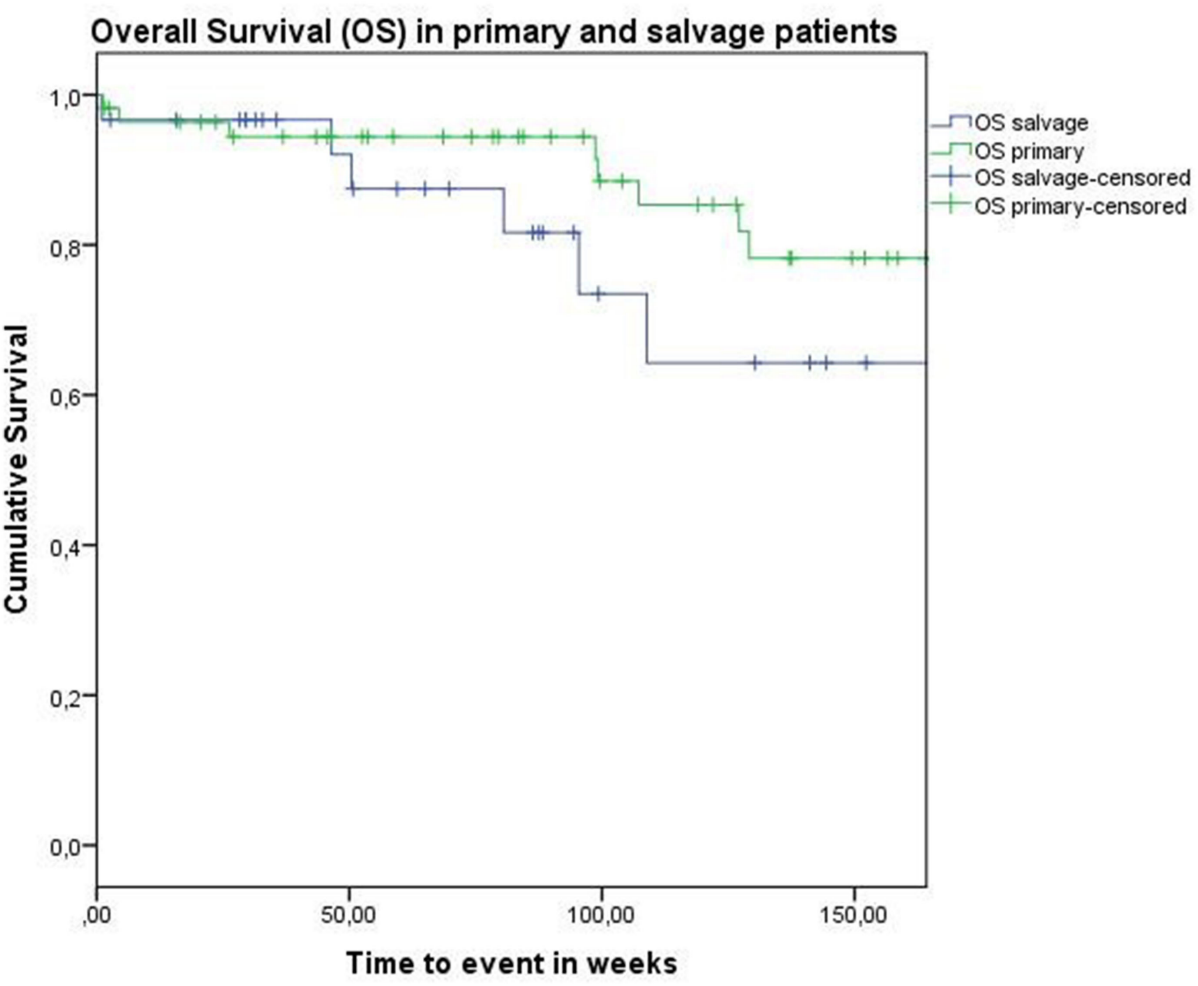
**Kaplan–Meier curve illustrating overall survival (OS) in patients treated with up-front or primary transoral robotic surgery (TORS) (green) and salvage TORS (blue)**. A trend toward better OS in the primary group is observed, although this difference is not statistically significant (log-rank test, *p* = 0.262).

**Figure 2:**
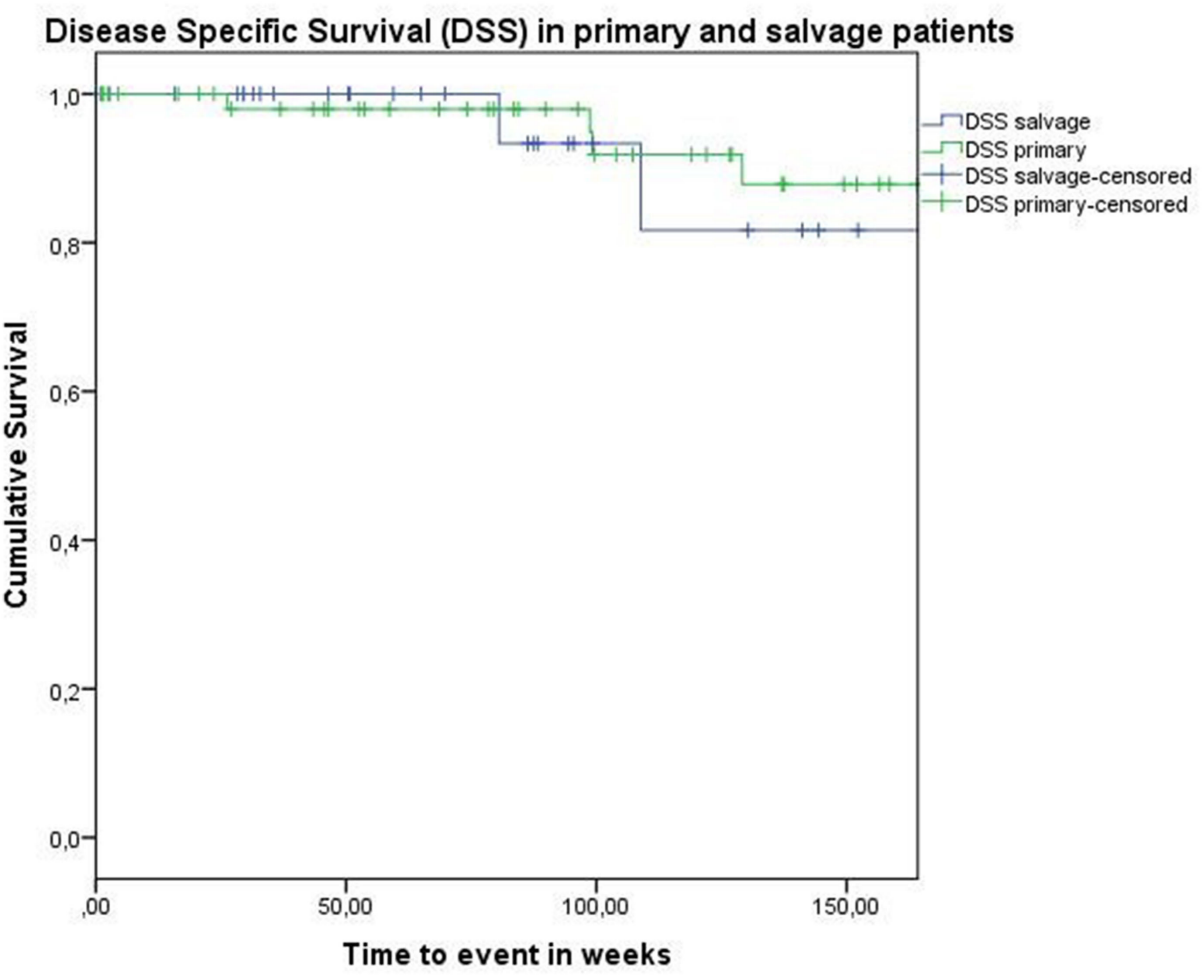
**Kaplan–Meier curve illustrating disease-specific survival in patients treated with up-front or primary transoral robotic surgery (TORS) (green) and salvage TORS (blue)**. No difference between survival curves is observed (log-rank test, *p* = 0.677).

**Figure 3:**
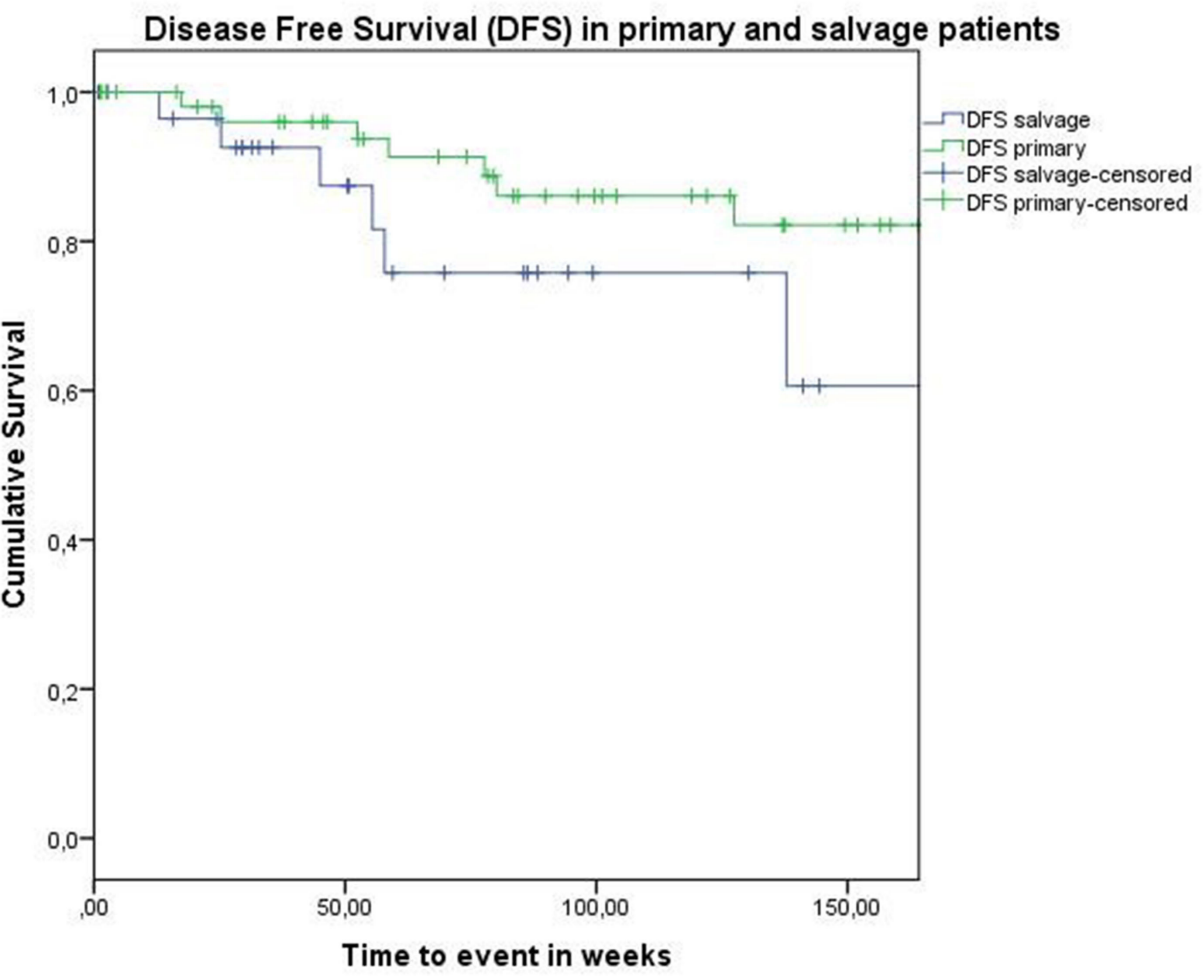
**Kaplan–Meier curve illustrating disease-free survival in patients treated with up-front or primary transoral robotic surgery (TORS) (green) and salvage TORS (blue)**. A trend toward higher disease control (locoregional and distant) in the primary treated group is observed, though without reaching a level of significance (log-rank test, *p* = 0.139).

Concerning disease control in the up-front group, one patient developed regional recurrence, one developed locoregional recurrence, and five patients were affected by distant disease. In the salvage group, two patients developed local recurrence, two regional recurrence, and two were affected by distant metastases. Looking at Kaplan–Meier survival estimates for the total population, estimated 2-year OS was 84.0% (SE = 4.8%), 2-year DSS was 92.0% (SE = 3.9%), and 2-year DFS was 82.8% (SE = 4.8%). Estimated 2-year OS in the primarily treated group was 88.5% (SE = 5.0%), while the estimated 2-year OS in the salvage group was 73.5% (SE = 10.9%) (Figure 1). However, this difference was not statistically significant (log-rank test, *p* = 0.262). Estimated 2-year DSS in the primary TORS group was 91.8% (SE = 4.6%) and estimated 2-year DSS in the salvage group was 93.3% (SE = 6.4%) (Figure 2). This finding did not reach statistical significance (log-rank test, *p* = 0.677). Estimated 2-year DFS in the primary TORS group was 86.1% (SE = 5.3%) and estimated 2-year DFS in the salvage group was 75.8% (SE = 9.7%) (Figure 3). As such, a trend toward higher disease control (locoregional and distant) in the primarily treated group was observed, though without reaching a level of significance (log-rank test, *p* = 0.139). The 2- and 3-year estimated survival rates are summarized in Table 4.

**Table 4:** **The 2- and 3-year survival estimates in the total population, the primary transoral robotic surgery (TORS) group, and the salvage TORS group**.

	2 years	3 years
OS total population	84.0% (SE = 4.8%)	74.2% (SE = 6.3%)
OS primary	88.5% (SE = 5.0%)	78.2% (SE = 7.1%)
OS salvage	73.5% (SE = 10.9%)	64.3% (SE = 12.8%)
DSS total population	92.0% (SE = 3.9%)	86.4% (SE = 5.3%)
DSS primary	91.8% (SE = 4.6%)	87.8% (SE = 5.9%)
DSS salvage	93.3% (SE = 6.4%)	81.7% (SE = 12.3%)
DFS total population	82.8% (SE = 4.8%)	76.5% (SE = 6.2%)
DFS primary	86.1% (SE = 5.3%)	82.2% (SE = 6.4%)
DFS salvage	75.8% (SE = 9.7%)	60.6% (SE = 15.6%)

*DFS, disease-free survival; DSS, disease-specific survival; OS, overall survival*.

## Discussion

Recently, TORS has been establishing itself as a valuable therapeutic adjunct in the treatment of head and neck cancer, and it is increasingly used as an alternative for conservative organ preservation treatments such as primary RT or CRT. Upon analysis of the National Cancer Database, Cracchiolo et al. discovered a steep rise of patients with low T-status (T1–T2) OPSCC who underwent primary surgical treatment. Of 8,768 patients in the USA with T1–T2 OPSCC, 68% underwent primary surgery, increasing from 56% in 2004 to 82% in 2013. FDA-approval of TORS treatment for early T-status OPSCC in 2009 is considered to be the main explanation for this shift in treatment strategy (23). Up to now, TORS is most frequently applied for primary or up-front surgery of OPSCC. In this setting, the TORS approach could prove advantageous for tailoring the adjuvant therapy strategy, which could not only result in downsizing adjuvant therapy but also in intensification depending on the definitive pathologic results. In a recent retrospective review of 76 patients who received TORS for OPSCC, up-front TORS deintensified adjuvant therapy: 72% of stage I/II patients avoided any adjuvant therapy and 46% of stage III/IV patients avoided CRT. Similarly, 52% of patients who would have received definitive RT as a single modality treatment could avoid it with primary surgery. On the other hand, definitive pathology after primary TORS resulted in intensification of adjuvant treatment in 33% of patients: they would have received RT alone based on clinical staging, but were treated with adjuvant CRT (24). In this study, the authors confirmed clinical N classification and overall clinical stage as significant factors in predicting the need for adjuvant therapy after TORS, suggesting that up-front TORS would be most beneficial for OPSCC patients with clinical N0 or N1 disease and those with clinical early-stage disease, making single modality treatment possible (24). However, in a cohort of 2,570 patients with cT1 to cT2 and cN0 to cN1 OPSCC receiving up-front surgery (including TORS) who were as such candidates for single-modality therapy, 28% of patients were pathologically T- or N-upstaged and in addition 33% had positive surgical margins and/or extracapsular spread, necessitating adjuvant RT or even CRT in 47% of patients (23). These findings stress the need for optimal treatment selection, avoiding upscaling of adjuvant treatment. The ability of up-front TORS to downsize adjuvant therapy is also illustrated by Lörincz et al. In their prospective cohort study, 40% of patients undergoing primary TORS did not need any adjuvant treatment and in another 34% of patients, adjuvant chemotherapy could be spared and adjuvant RT could be reduced (25). In the currently largest multi-institutional retrospective review, including 410 patients with head and neck cancer primarily treated by TORS, adjuvant treatment could be avoided in 39% of cases (26). However, a major drawback of this study was the multi-institutional setup, resulting in non-standardization of the adjuvant therapeutic strategy. In our primary surgery study population (*N* = 56), any adjuvant therapy was not indicated in 23.2% of patients, while 26.8% did receive trimodality treatment. Adjuvant RT proved necessary in 46.4%.

Concerning oncologic outcomes after up-front TORS, we reported an estimated 2-year OS, DSS, and DFS of 88.5, 91.8, and 86.1%, respectively. This is comparable to the 2-year OS and DSS of 91 and 94.5% as reported by de Almeida et al. (26) and the 2-year OS of 89.3% as reported by White et al. (27). Lörincz et al. reported an OS rate of as high as 94%, but with a recurrence-free survival rate (including distant disease and as such identical to our definition of DFS) of 80% (25). In our group of patients who received TORS as a salvage treatment (*N* = 30), a 2-year OS, DSS, and DFS of 73.5, 93.3, and 75.8% were reported, respectively. These results are comparable to the 2-year OS and recurrence-free survival of both 74% as reported in a retrospective multi-institutional case-control study of 64 patients who underwent salvage TORS and 64 patients receiving salvage open surgical approaches for recurrent OPSCC (19). Although we observed a trend toward higher DFS and OS in the up-front TORS group when compared to the salvage TORS group, these differences did not reach statistical significance upon univariate analysis, confirming TORS as a valuable alternative to salvage open en bloc surgery. These data clearly prove the feasibility and favorable outcomes of TORS in this difficult to treat salvage group.

Concerning function preservation after TORS, excellent results were obtained in our study population. A total of 69.1% of up-front TORS patients and 40% of salvage TORS patients were able to resume an oral diet immediately after the operation. As one can expect, swallowing outcomes in terms of G-tube dependency seem favorable in the up-front TORS group (1.8% G-tube dependency) when compared to the salvage group (20% G-tube dependency). No definitive tracheotomies were reported. These functional outcomes are comparable to the data in the literature: in a recent systematic review on the topic, Yeah et al. identified among 13 TORS studies a feeding-tube dependency rate ranging from 0 to 20.7% at 1-year postoperative follow-up. The rate of tracheostomy dependence among nine TORS studies ranged from 0 to 3.5% at 2 years postoperative follow-up (28).

An important issue to address is whether these oncologic and functional results are favorable or at least similar to the results obtained by organ preservation strategies. In the aforementioned systematic review (28), the authors concluded that, because no randomized trials comparing TORS versus intensity-modulated radiotherapy (IMRT) based regimens are currently available, only uncontrolled reports suggest comparable oncologic outcomes with TORS compared to IMRT of OPSCC, while functional outcomes may be superior after TORS. This conclusion needs to be interpreted with caution, as TORS studies included patients with earlier stage OPSCC when compared to the IMRT population, representing an important selection bias. On the other hand, mean follow-up of TORS studies is short when compared to conservative organ preservation studies, possibly favoring the definitive functional outcome after TORS. Moreover, direct comparisons of swallowing outcomes between patients treated with any kind of transoral surgery, including TORS, and non-surgical treatment is not possible because of the varying methodology and heterogeneity of swallowing outcome measures used (29). In this respect, the presumed functional superiority of TORS versus non-surgical treatments remains to be critically appraised until level I evidence will be available. Several surgical trials have recently begun to determine the effectiveness of transoral management of OPSCC, e.g., the ECOG3311 phase III trial comparing oncologic efficacy in surgical versus non-surgical approaches to OPSCC, or the ORATOR phase II trial, comparing TORS to radiation with or without chemotherapy with quality of life as primary endpoint after 1 year (30). Of interest, a very recent retrospective stage-matched cohort study comparing OS and DSS and G-tube prevalence between 88 patients with OPSCC treated with CRT and 39 patients treated with TORS reported an indistinguishable survival but lower gastrostomy prevalence in the surgical group (31).

There are some limitations to our study. As a retrospective study, we are dealing with an inherent selection bias. Moreover, being a multi-institutional study, no standardization of decision-making, staging, operative procedure, and adjuvant therapy can be guaranteed. However, all management decisions were made during multidisciplinary tumor board meetings. Moreover, because of the small study population and small subgroups, multivariate analysis was not possible. Moreover, we were confronted with high rates of adjuvant RT or CRT in the up-front TORS group. This can partly be explained by selection of cT3–T4 and cN2 patients for primary surgery. On the other hand, high rates of close and positive surgical margins necessitated adjuvant therapy. Presumably, these high rates of involved margins are related to the non-systematic use of frozen sections to account for resection radicality after TORS tumor removal on the one hand and to pathology assessment-related issues on the other hand. Regarding the latter issue, interpreting margins after electrocautery resection during TORS is clearly difficult due to coagulation artifacts. This may be partly overcome by using the carbon dioxide laser, but at the moment of the study none of the surgical departments disposed of a Da Vinci mountable flexible laser fiber. This laser resection theoretically would lead to better evaluable margins, but has the disadvantage of much more difficulties in controlling hemorrhage. Furthermore, small tumor specimen ruptures during TORS are not unusual due to manipulation by the Maryland forceps and could be misinterpreted by the pathologist as being a compromised margin. This finding stresses the need for cautious tumor manipulation and optimal communication with the pathologist. As a quality reinforcing measure to cut down future margin involvement, a new protocol for specimen orientation after resection, allowing for optimal margin assessment by the pathologist, has been introduced. Additionally, use of frozen sections in all TORS patients is heavily advocated among the participating centers. Another possible additional explanation of the high positive surgical margin rate is that this case series reports the first cases in all three participating centers and as such includes patients at the beginning of the learning curve. Moreover, the rather low yearly case load (86 cases during 6 years in three centers) also possibly influences outcome. This also has to do with (1) a high experience with the cheaper technique of TLM and (2) the limited availability of the operating robot due to a high frequency of use by other departments, i.e., urology and gynecology.

## Conclusion

This multi-institutional retrospective case series confirms favorable oncologic and functional outcomes of TORS for selected head and neck malignancies, both in the primary and in the salvage setting. Although we observed a trend toward higher DFS and OS in the up-front TORS group when compared to the salvage TORS group, these differences in oncologic outcome did not reach statistical significance. This finding supports TORS as a valuable alternative to salvage open en bloc surgery and confirms its additional role in the treatment of this difficult to treat salvage patient group.

## Author Contributions

JM was involved in conceiving the project, Ethical Committee approval, data collection and analysis, and drafting and revising the manuscript. CV and TV were involved in data collection, patient care, and critically reviewing the manuscript. ED was involved in data collection and critically reviewing the manuscript. SN and PC were involved in conceiving the project, Ethical Committee approval, and revising the manuscript. RH and PD were involved in conceiving the project, Ethical Committee approval, patient care, and revising the manuscript. VV was involved in conceiving the project, Ethical Committee approval, data collection and analysis, patient care, and drafting and revising the manuscript.

## Conflict of Interest Statement

The authors do not have any potential conflict of interest to declare in relation with the content of this article.
